# Micellization of a starch–poly(1,4-butylene succinate) nano-hybrid for enhanced energy storage†

**DOI:** 10.1039/d1ra00635e

**Published:** 2021-03-22

**Authors:** O. D. Saliu, M. A. Mamo, P. G. Ndungu, J. Ramontja

**Affiliations:** Energy, Sensors and Multifunctional Nanomaterials Research Group, Department of Chemical Sciences, University of Johannesburg P. O. Box 17011 Doornfontein 2028 Johannesburg South Africa messaim@uj.ac.za jamesr@uj.ac.za pndungu@uj.ac.za

## Abstract

In this work, we report on a reverse micellization approach to prepare uncarbonized starch and poly(1,4-butylene succinate) hybrids with exceptional charge storage performance. Uncarbonized starch was activated through protonation, hybridized with poly (1,4-butylene succinate), configured into conductive reverse micelles, and incorporated with magnetite nanoparticles. Before magnetite incorporation, the maximum specific capacitance (*C*_sp_), energy density (*E*_d_), power density (*P*_d_) and retention capacity (%) of the reverse micelles were estimated to be 584 F g^−1^, 143 W h kg^−1^, 2356 W kg and 97.5%. After magnetite incorporation, we achieved a maximum supercapacitive performance of 631 F g^−1^, 204 W h kg^−1^, 4371 W kg^−1^ and 98%. We demonstrate that the use of magnetite incorporated St–PBS reverse micelles minimizes the contact resistance between the two supercapacitor electrodes, resulting in high charge storage capacity.

## Abstract 2

In this work, we report on a reverse micellization approach to prepare uncarbonized starch and poly(1,4-butylene succinate) hybrids with exceptional charge storage performance.

## Graphical abstract

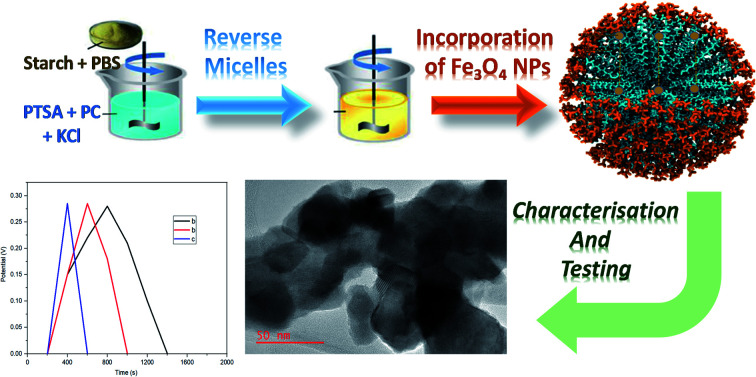

## 1. Introduction

The process of employing energy systems to generate, convert and store energy needs to meet various sustainability indices, and the energy systems used should meet environmental, economic and social quality requirements.^1^ One central tenet, which any sustainable energy system must meet, is that it must provide accessible energy to the present generation without affecting future generations.^2^ This can only be possible when the energy systems are built from materials that are less harmful (from cradle to grave), easily available, and relatively inexpensive. Conventional fossil fuels can be difficult to handle, are not renewable, disrupt local ecologies, are associated with various geo-political problems, pollute the environment and are increasingly being depleted at faster rates.^3,4^ Solar, wind, biofuels, hydrothermal are examples of renewable and sustainable energy systems that are presently gaining significant worldwide attention. However, the energy generated from these renewable resources must be stored in portable or stationary devices to ensure cleaner and smarter, and more accessible energy.^5,6^

Various electrodes that promise to meet various sustainability goals for energy storage include carbon nanomaterials from biomass such as coconut husks, bamboo, sugar-cane, banana peels, cocoa pods, rice husks, almonds, palm kernels and many more.^7–10^ These carbon based electrodes have also been obtained from fish gills, corn syrups; but there has been limited research directed towards biopolymers like starch, poly(lactic acid), alginates, cellulose, chitosans and many more, for energy storage in supercapacitors. The few works reported on the use of starch, cellulose and other biopolymers shows that they are first converted to carbon forms before they are utilised in energy storage devices. This principle is a bit debatable, since carbonization itself makes use of high temperature and can utilize harsh solvents, which reduces the overall sustainability potential of the biopolymer materials in the long-run.

This work designed and implemented a unique synthesis and assembly protocol to develop an enhanced synergy between ‘uncarbonized’ biopolymers and nanomaterials to fabricate sustainable supercapacitor electrodes. The idea behind this work, is that the functional groups on the selected biopolymer can link easily with nano-oxides of transition metals to obtain suitable physico-chemical properties that are viable for energy storage. The use of uncarbonized starch portrays good sustainability and leverage on the biocompatibility, functionality and renewability of starch.^11–13^ Apart form the metal oxides, other hybrids of natural polysaccharides with several biodegradable polymers and conductive polymers have been reported.^14–17^ Conductive polymers like poly(3,4-ethylenedioxythiophene), polyaniline, and polypyrrole have shown similar or better pseudocapacitve capabilities than metal oxides for energy storage applications.^18–22^

The uncarbonized starch was activated through protonation to improve its electrochemical properties, and hybridized with poly(1,4-butylene succinate) (PBS), a semicrystalline biopolymer to make a hybrid biopolymer–polysacharide composite. The hybrids formed were configured into conductive reverse micelles, starch formed the head and PBS formed the tail.^23^ Micelles or reverse micelles attains spherical, ellipsoidal, cylindrical and bilayer conformations, and different conformations shows different diffusive, conductive and ionic properties,^24–26^ which may affect their electrochemical properties in super-capacitor applications. In addition, different micellar designs or conformations show differing surface charge distribution that stabilizes their cores.^24,27^ The three different reverse micelles designed in this work showed different supercapacitve properties and magnetite nanoparticles were then incorporated within their cores to enhance free flow of ionic charges.

There are very few works on the application of micelles for supercapacitor applications. However, there are numerous reports on the use of carbon aerogels, materials that can be synthesised using micelle based soft templates, which have not produced very high specific capacitances and energy densities. Liu *et al.*, reported on the design of nitrogen-doped carbon based non-nano micellar structures with specific capacitance of 271 F g^−1^. Sun *et al.*, also reported on the synthesis of carbon aerogels that rearranges into spherical micelles with tunable porosity in the presence of [C_16_Im]BF_4_. The specific capacitance obtained was 188 F g^−1^, with a corresponding energy and power densities of 9.08 W h kg^−1^ and 6250 W kg^−1^. Till now, and to the best of our knowledge, no public research has so far been published on the use of the micellar forms of uncarbonized starch and PBS nano-hybrids for applications in supercapacitors.^20^

Different micelles orients in a way to increase their overall ionic stability. By carefully positioning metal oxides within micellar forms of uncarbonized biopolymer, excellent charge transfer kinetics can be achieved, and this can form the basis of how starch can be used as a supercapacitor electrode without converting it into a carbon form.^28,29^ The switching of the conformations of these micellar nano-architectures can therefore be used to tune the conductance, capacitance, energy and power densities of supercapacitor electrodes for the storage of electrical charges.^28–30^ For example, this method is applied in conductive electrophoretic image displays, electrorheological fluids, and ink jet imprintings.^29–32^

Therefore, the aim of this particular work is to primarily use a green method to specifically design different sustainable micellar nano-architectures based on poly(1,4-butylene succinate) cores, and uncarbonized starch heads with impregnated nano-magnetite. The effect of each micellar design on the overall electrochemical conductivity and capacitive performance of starch–PBS nanohybrids will be studied. The novelty of our work is the use of different micellar designs to tune the supercapacitve properties of an uncarbonized starch based supercapacitor electrode.

## 2. Materials and methods

Materials used for this work; which include corn starch, poly(1,4-butylene succinate) (PBS) extended with 1,6-diisocyanato hexane, ammonium persulphate, ferric chloride hexa-hydrate (FeCl_3_·6H_2_O), ferrous chloride tetra-hydrate (FeCl_2_·4H_2_O), glass fibre separator, nickel foam, platinum wires, citric acid, *p*-toluene sulphonic acid, ammonium chloride, potassium chloride, acetonitrile, isopropanol, aqueous ammonia, oleic acid, Tween-80, dichloromethane (DCM), tergitol were obtained from the local supplier for Sigma Aldrich Pty Ltd.

### 2.1. Preparation of activated starch–poly(1,4-butylene succinate) hybrid (St–PBS)

Briefly, a mixture of 30 mL of starch and 5 mL of citric acid was formed by taking each proportions from 10% of starch and 2% citric acid in grams per millilitres. The mixture was stirred at 70 °C for one hour on a magnetic stirrer to obtain an homogenous slurry. An activating solution was synthesized by mixing 0.5 g of *p*-toluene sulphonic acid, dissolved with 15 mL each of 1 M ammonium chloride, 0.5 M potassium chloride and 15 mL propylene carbonate; under overnight reflux at 50 °C and 250 rpm. The starch–citric acid mixture was poured into the prepared activating solution and stirred under liquid nitrogen gas for one hour at 60 °C, and a nitrogen flow rate of 200 mL min^−1^. After the mixture settles, 30 mL from a PBS solution prepared by dissolving PBS in acetonitrile solvent to form a 10% concentration was introduced into the activated starch colloidal solution. The activated starch and PBS mixture was mixed in the presence of 0.001 M ammonium persulphate for 4 hours at 100 °C on a magnetic stirrer. The resulting product was removed by centrifugation, washed with ethanol, filtered and air dried.^20,23^

### 2.2. Synthesis of magnetite nanoparticles

The magnetite nanoparticles were synthesized using the co-precipitation method with slight modification. 20 mL of 0.0125 M FeCl_3_·6H_2_O and 10 mL of 0.0125 M FeCl_2_·4H_2_O were dissolved in 30 mL isopropanol on a magnetic stirrer at 300 rpm for 25 minutes. A stabilizing solution was prepared alongside by making a 4% oleic acid solution in glycerol. The ferric and ferrous ion mixture was introduced into the stabilizing solution and stirred for 30 minutes at room temperature. 8 mL of ammonium hydroxide was introduced dropwise into the stabilized precursor mixture and stirred further for 40 minutes at 400 rpm and 50 °C until a pH of 10 was attained. The resultant black precipitate was washed, filtered and dried in an oven at a temperature of 50 °C for 6 hours. The method was adapted from the ref. 33.

### 2.3. Preparation of various (St–PBS) micellar architectures

Starch and poly(1,4-butylene succinate) were made into three different types of reverse micelles and labelled as St–PBS micelle I, II and III. The first reverse micelle was designed using the precipitation method. In this method, 1.2 g of the prepared St–PBS hybrid was dissolved in 15 mL acetonitrile under room temperature using a magnetic stirrer at 250 rpm. The mixture was introduced into 25 mL of 5% tween-80 solution and further stirred for 6 hours at 90 °C, to ensure the complete evaporation of acetonitrile. The obtained gel was pre-set in a refrigerator at 4 °C for 24 hours, vacuum-dried under a pressure of 720 mmHg for 5 hours and weighed. The drying was done until two consecutive weights matched; indicating equilibrium was reached within the reverse micelle.

The second reverse micellar design was designed using the single emulsion technique. In this technique, 1.2 g of the prepared St–PBS hybrid was dissolved in 20 mL, 1 : 1 dichloromethane and acetonitrile, under room temperature using a magnetic stirrer at 250 rpm. The mixture was introduced into 25 mL of 5% tween-80 solution and further stirred for 6 hours at 90 °C, to ensure the complete evaporation of acetonitrile and dichloromethane. The mixture was pre-set and dried under the same condition for the first reverse. The third micellar design was fabricated using the double emulsion method. Samples were typically prepared by dissolving 1.2 g of the prepared St–PBS hybrid was dissolved in 20 mL, 1 : 1 dichloromethane and acetonitrile, under room temperature using a magnetic stirrer at 250 rpm. The mixture was introduced into 15 mL of 5% tween-80 and 10 mL of 3% tergitol and stirred for 6 hours at 90 °C, to ensure the complete evaporation of acetonitrile and dichloromethane. The mixture was pre-set and dried under the same condition for the first reverse. All methods were modified and adopted from the ref. 34 and ^35^.

### 2.4. Preparation of St–PBS-magnetite micellar nanocomposites

A weighed amount (0.1 g) of the prepared St–PBS micelle I, II and III were differently dispersed in 5% solution of tetrahydrofuran and *p*-toluene sulphonic acid on a magnetic stirrer at 70 °C for 30 minutes, to completely reach homogeneity. The acidity of the mixture was adjusted to reach a pH of six. Immediately, 0.05 g of magnetite nanoparticles was introduced into the mixture and stirred for 60 minutes in a three-necked flask, at 50 °C under reflux. Powder products were obtained through centrifugation, washed and pre-set under refrigeration at 4 °C. The final product was air dried.^36^

### 2.5. Characterization

Samples were analysed on a Fourier Transform Infrared (FTIR) Spectroscope equipped with an ATR accessory (Spectrum-100 PerkinElmer, USA). The FTIR analysis was carried out in the wave number range of 4000 to 600 cm^−1^. X-ray diffraction (XRD) patterns were obtained on a Rigaku Ultima IV X-ray diffractometer, employing CuKα radiation at a wavelength of 1.5406 Å (generated at 45 kV and 40 mA). XRD patterns were collected in the 2*θ* range between 5° and 90° with a step size of 0.01°, and a scan speed of 1° min^−1^. The particle sizes of the nano-hybrids were investigated using a Dynamic Light Scattering (DLS) instrument; Zetasizer, model ZEN 3600. The morphologies of the samples were determined using scanning electron microscopy (SEM), (TESCAN, VEGA SEM, Czech Republic) at a 20 kV electron acceleration voltage. The surfaces of the samples were coated with carbon to avoid charging.

### 2.6. Conductivity and electrochemical measurements

The conductivities of the St–PBS micelles were determined using a four-probe system, while their electrochemical characteristics were investigated on a Gamry potentiostat in both three and two cell configurations. In the three cell configuration, a platinum wire, an Ag/AgCl (saturated by 3 M NaCl), and St–PBS-magnetite electrodes on nickel foam were used as the counter, reference, and working electrode in 1 M Na_2_SO_4_ electrolyte, respectively. The working electrode was prepared by loading on nickel foam; a 10 mg mixture of St–PBS micelles and poly(vinylidene difluoride) powder dissolved in *N*-methyl pyrrolidone in 80 : 20 by weights. The loadings were achieved through doctor blading, using a specially designed smooth and thin glass. Cyclic voltammetry (CV) and galvanostatic charge–discharge (GCD) measurements were performed with potentials ranging from 0 to 0.7 V. Electrochemical impedance spectroscopy (EIS), was conducted over a frequency range of 100 kHz to 0.1 Hz with perturbation amplitude of 0.01 V.^37–40^ In the two-cell configuration, which involves the working electrode and counter electrode set-up, a glassy fibre separator was soaked in 1 M Na_2_SO_4_ electrolyte, sandwiched in between two symmetrical working electrodes. The two-cell working electrodes were prepared using the same method employed for the three-cell working electrodes. The symmetrical supercapacitor was aligned, pressed and clipped before setting it up for two-cell electrochemical measurements.

## 3. Results and discussion

The St–PBS reverse micelles with high charge storage capacity were designed from starch, and poly(butylene succinate) biopolymers. The starch provides porous channels for easy transfer of charges within the micelles,^41^ while PBS improves the recyclability of the micelles and help the micelle to store charges over a wide temperature window.^42^

### 3.1. FTIR and XRD analysis of the starch–PBS samples

**Fig. 1:**
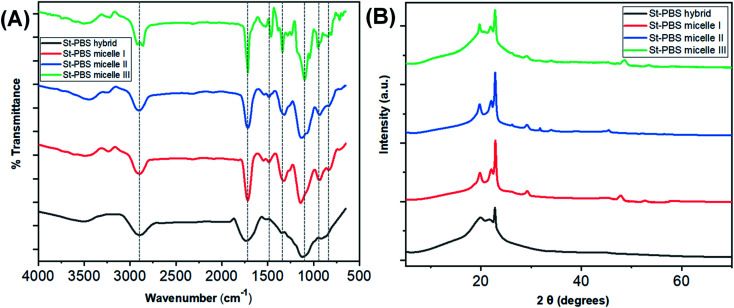
The FTIR spectra of the St–PBS samples are presented in image (A) and the XRD diffraction patterns are presented in image (B).

Fig. 1(A) shows the FTIR spectra of starch–PBS micelle I, II, III and ordinary starch–PBS hybrid. The main differences among the three reverse micelles are observed in the absorption bands, 1800–700 cm^−1^. The successful design of the reverse micelles was confirmed from the differences between the OH vibration regions of the St–PBS hybrids before and after micellization. Before micellization, the St–PBS hybrids showed a diminished O–H band, broad C–H bands at 3430 cm^−1^ and 2870 cm^−1^.^43^ The intensities of bands at 1169, 1085 and 920 cm^−1^ attributed to C–O and C–C stretch with C–O–H contributions. The C=O adsorption band at 1710 cm^−1^ for PBS narrowed after micellization, indicating strong bond formation between the starch and poly(butylene succinate) polymers.

After micellization, the O–H stretching vibrations of St–PBS micelles I, II and III shifted to 3502, 3505 and 3511 cm^−1^. Between 1300–900 cm^−1^, C–O–C glucosidic bonds, C–O–H bending and stretching vibrations, and C–O stretches were observed, and duplet C–H bends at 810 and 802 cm^−1^. St–PBS micelles I and II showed a more resolved band than St–PBS micelle III around 1050 to 950 cm^−1^; suggesting that St–PBS micelles I and II have shorter-ranged double helices than micelle III.^44^ Changes and shifts in the peak positions between 1300–900 cm^−1^ can be used to probe interactions between the starch and PBS.^40,44,45^ In general, the peaks shifted to slightly lower wavenumbers when comparing St–PBS micelles I (1323, 1141, and 937 cm^−1^) and II (1319, 1129, and 933 cm^−1^), which suggests an increase in hydrogen bonding between the starch and PBS components.^46^

The XRD analysis of the St–PBS hybrids (Fig. 1(B)), revealed diffraction patterns at 2*θ* angles of 19.7°, 22.2°, 26.34° and 29.10°, which are attributed to the (020), (110), (1̄21), and (111) reflections from the α crystal of the PBS component within the hybrids.^47^ When mixing starch and PBS, some authors have noted that the low intensity and relatively broad peaks of the starch molecules will not be observed in the composite, due to the mixing of the starch and with the much more crystalline PBS.^23^ However, we did observe some minor diffraction peaks in the three starch micelles composites, St–PBS micelle I–III, from 46° to 53°, which suggests the micellization process improves the crystallinity of the composite. From the most intense 2*θ* peak at 29.10°, the ‘*d*’ spacings from Scherrer equation for the St–PBS micelle I–III samples were 23.24, 19.38 and 26.17 nm respectively. The *d*-spacing values confirmed that the St–PBS micelle samples were relatively well-ordered with some crystallinity in the structures, and domains are in the nanometre range.

### 3.2. Contact angle, and conductivity determination of the starch–PBS samples

The three reversed micelles showed contact angles of 72.49°, 74.81° and 75.01°, which suggests good wetting properties.^48^ The three reversed micelles had surface tensions of 50.23 ± 0.2, 58.14 ± 0.2 and 62.69 ± 0.1 N m^−1^; implying their surfaces are favourable for wetting and will coat well on the current collectors, and should allow for excellent ingress of the electrolytes.

**Fig. 2:**
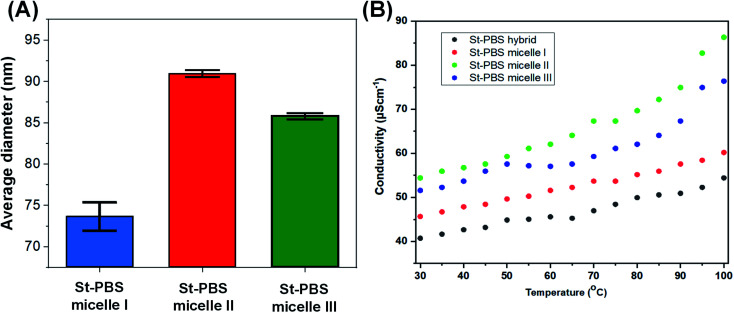
Image (A) presents the average size of the St–PBS reverse micelle samples determined using DLS. Image (B) presents the results of the conductivity measurements of the various St–PBS samples.

The average size of the St–PBS reverse micelle samples varied from 70–90 nm, with no apparent trend in the variation of the sizes as determined by DLS (Fig. 2(A)). The conductivity of the reversed micelles increased with temperature (Fig. 2(B)), with micelle II showing the highest conductivity. A conductivity value of 76.13, 84.97 and 59.77 S cm^−1^ was obtained at 373 K, for St–PBS micelle I, II and III respectively. This implies that the St–PBS micelles II sample should possess better capacitive properties, and maybe more stable when voltage is applied during electrochemical testing. Temperature increase creates distortion, which in turn, creates polarons and bipolarons movement within the chains of the starch micelles that had already been doped with *para*-toluene sulphonic acid. The flow of polarons and bipolarons is what is responsible for the conductivity of the designed micelles.

### 3.3. SEM analysis of the starch–PBS samples

**Fig. 3:**
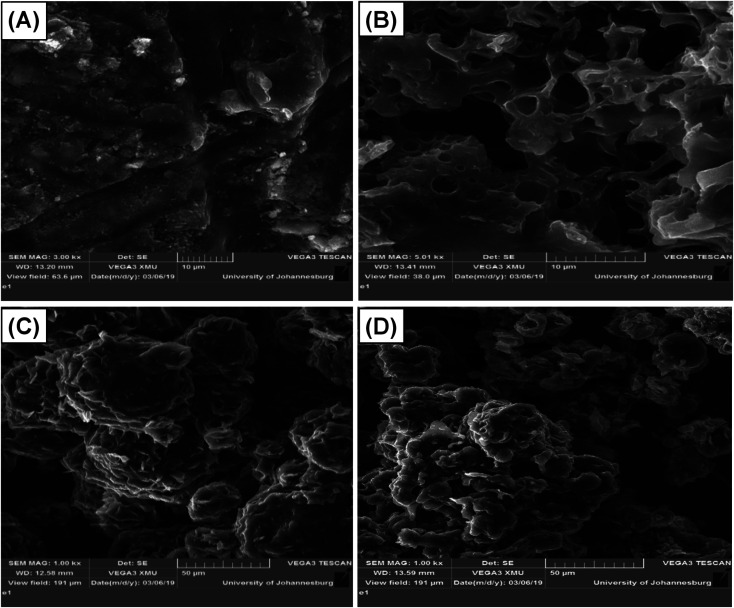
SEM of (i) ordinary St–PBS hybrid, (ii) St–PBS micelle I, (iii) St–PBS micelle II and (iv) St–PBS micelle III.

The SEM images presented in Fig. 3 revealed that, before micellization, the St–PBS hybrid showed a rubbery-like surface with irregular shallow channels. After micellization, the St–PBS micelle I appeared glassy, with a hollow and porous microstructure, the St–PBS micelle II had agglomerated structures of irregularly shaped particles with some porosity, and the St–PBS micelle III also appeared to have randomly shaped agglomerated structures, with some spherical particles and porosity. The prominent pores seen in the St–PBS micelle II sample may be because of the precipitation method employed in its fabrication, while the irregular morphology of St–PBS micelle III may be due to effect of competing surfactants used in its synthesis.^40,49^ The differences in morphology are expected to have an effect on the supercapacitor properties due to differences in charge transfer and storage.

### 3.4. Electrochemical measurements on starch–PBS samples

#### 3.4.1. Electrochemical measurements on starch–PBS sample using a three electrode configuration

**Fig. 4:**
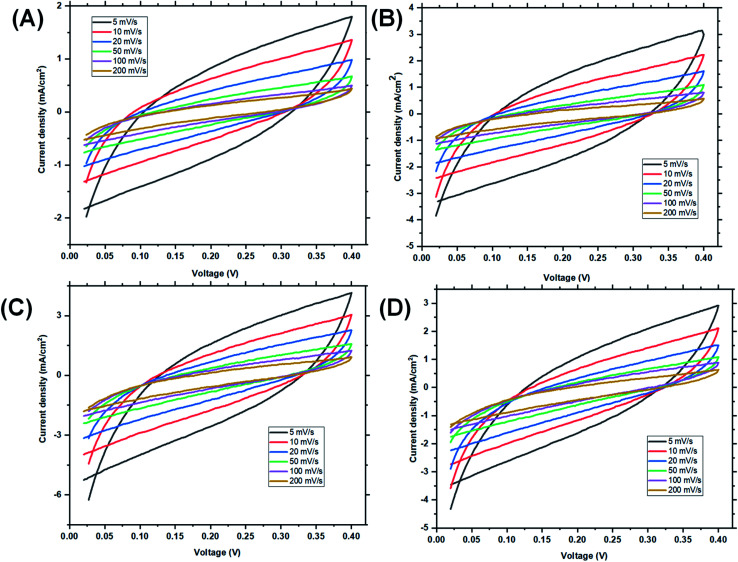
Cyclic voltammetry (CV) of ordinary St–PBS hybrid (A), St–PBS micelle I (B), St–PBS micelle II (C), and St–PBS micelle III (D). The voltammograms were acquired using a three electrode configuration, with a platinum wire counter electrode, an Ag/AgCl reference electrode, and the St–PBS materials loaded on nickel foam.

Cyclic voltammetry (CV) was used to investigate the capacitive behaviours of the three reverse micelles designed. The specific capacitances (*C*_sp_) of the hybrids before and after micellization were calculated from the areas of CV curves (Fig. 4). At scan rates of 200, 100, 50, 20, 10 and 5 mV s^−1^, St–PBS had a *C*_sp_ of 42, 81, 122, 187, 236, and 301 F g^−1^. While the St–PBS micelle I had *C*_sp_ of 49, 89, 143, 195, 250, and 324 F g^−1^, St–PBS micelle II showed *C*_sp_ of 75, 129, 247, 391, 483, and 583 F g^−1^, and St–PBS micelle III showed *C*_sp_ of 48, 88, 131, 190, 243, and 318 F g^−1^. All the CV curves, for the ST-PBS and St–PBS micelles' I–III, were quasi-rectangular in shape, which is typically observed with materials exhibiting supercapacitive behaviour.^50,51^ In addition, the CV curves did not show any redox peaks, and thus highlights that the energy stored in the micelles were based purely on electrostatic and surface diffusion mechanisms. The St–PBS micelle II showed highest *C*_sp_ among the three reverse micelle structures.

At high scan rates, diffusion resistance and polarization phenomena increase, and hence the *C*_sp_ is low. The *C*_sp_ values of 324 and 584 F g^−1^ of St–PBS micelles I and II at 5 mV s^−1^ are currently the highest specific capacitances for any un-carbonized starch based supercapacitor. Zhou *et al.*^52^ obtained a *C*_sp_ of 195 F g^−1^ at 1 A g^−1^ using starch derived, mesoporous carbon spheres, Cao *et al.*^41^ used MnO_2_ to create pores in hierarchical starch based carbon, a *C*_sp_ of 229 F g^−1^ was obtained at 1 A g^−1^ in 6 M KOH electrolyte. Past work, in the open literature, typically reports on the use of carbonized starch for supercapacitor electrodes, while our work reports on the use of un-carbonized starch micelle structures. Furthermore, the starch–PBS micelle structures were synthesized with a simple and relatively environmentally friendly approach to achieve a very high specific capacitance. In contrast to our work, one of the earliest reported work on use of un-carbonized biopolymers, where Jiao and his team fabricated un-carbonized cellulose nano-fibrils, *C*_sp_ and energy density of 81.3 F g^−1^ and 2040 W kg^−1^ were obtained; which is lower than our reported *C*_sp_ and energy densities.^53,54^ A few other comparable examples in the literature include the use of wood PANI composites (maximum *C*_sp_ of 304 F g^−1^),^55^ development of polypyrrole cellulose hydrogels (maximum *C*_sp_ of 255 F g^−1^),^56^ and a PEDOT lignin poly(aminoanthraquinone) composite (maximum *C*_sp_ of 418 F g^−1^).^57^

**Fig. 5:**
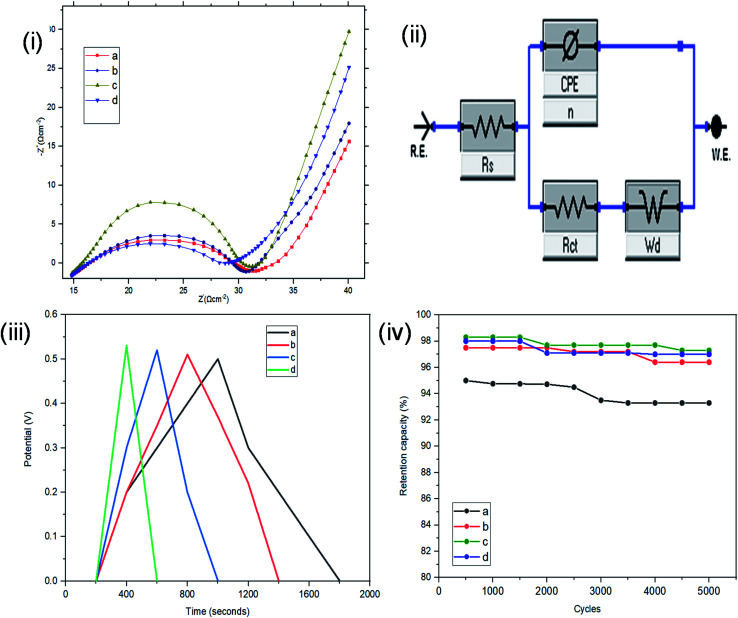
Three-electrode configuration: (i) EIS of ordinary St–PBS hybrid, St–PBS micelle I, St–PBS micelle II and St–PBS micelle III (a–d). (ii) Constant phase element (CPE); equivalent circuit model of the EIS (iii) GCD of ordinary St–PBS hybrid, St–PBS micelle I, St–PBS micelle II and St–PBS micelle III (a–d). (iv) Retention capacity of ordinary St–PBS hybrid, St–PBS micelle I, St–PBS micelle II and St–PBS micelle III (a–d).

Resistance, charge transport and frequency response of the ST–PBS micelles were investigated using EIS. Properties like solution resistance (*R*_s_), charge transfer resistance (*R*_ct_), and Warburg impedance (*W*) were obtained from Nyquist plots of the micelles (Fig. 5(i)). The St–PBS micelle I had an *R*_ct_ of 16.81 Ω, St–PBS micelle II showed an *R*_ct_ of 13.5 Ω and St–PBS micelle III showed an *R*_ct_ of 14.25 Ω. The vertical line at low frequency region, which tilt more towards *y*-axis suggests good low diffusion resistance and good supercapacitive behaviour. The ‘*n*’ value of 1 indicates ideal capacitors, while a value of 0 indicates ideal insulators. ‘n’ values of 0.55, 0.68, 0.59 for St–PBS micelles I, II and III indicates they have ability to be employed for supercapacitive applications. ‘*n*’ value of 0.39 was observed for ordinary St–PBS hybrids, which implies a more insulator-like behaviour.^58,59^ The EIS was further modelled using constant phase element (CPE) equivalent circuit (Fig. 5(ii)), whose impedance depend on *n*th power of frequency, and CPEs of 0.147, 0.143 and 0.149 were observed for the St–PBS micelles I, II and III.

Symmetrical and triangular curves were observed with the GCD measurements of the reverse micelles in Fig. 5(iii). The GCD curves show how applied voltage varies with time of charge and discharge, and the symmetry and shape of the curves illustrate that the charge storage mechanism conforms to that involving an electric double layer at the interface of the electrode materials and electrolyte.^60–62^ The slight changes in symmetry from the ordinary St–PBS hybrid (curve a), St–PBS micelle I (curve b), St–PBS micelle II (curve c) and to the St–PBS micelle III (curve d), highlights how the synthetic method improved the ideal capacitive behaviour of the materials. All of the three St–PBS reverse micelles completed their charge–discharge cycles faster than the ordinary St–PBS hybrid. The ordinary St–PBS hybrid completed its GCD cycle after 1400 seconds while St–PBS micelles I, II and III completed theirs in shorter periods of 800, 600 and 400 seconds respectively. This highlights that the micellization process not only affects the morphology, as seen with the SEM images, but also improves the charge and discharge kinetics of un-carbonized starch. The retention capacity of the St–PBS hybrid (Fig. 5(iv)) was 93%, and it improved after micellization process, with St–PBS micelle I having a retention capacity of 96.5, and St–PBS micelles II and III having 97.0 and 97.5% respectively.

The energy densities of the St–PBS hybrids are presented in Table 1, and in general, the values decreased as the scan rate increased. Before micellization, the maximum value for the energy density (*E*_d_) of the St–PBS was 73 W h kg^−1^, at a scan rate of 5.0 mV s^−1^. After micellization, the St–PBS micelles I, II and III showed maximum values of 79, 143 and 77 W h kg^−1^ respectively at a scan rate of 5.0 mV s^−1^. The corresponding power densities for the ordinary St–PBS was 1640 W kg^−1^, and the St–PBS micelles I, II and III had power densities of 2118, 2356, 2254 W kg^−1^ respectively. Discussions on the significance of these results are presented in comparison to the samples tested in a two electrode cell configuration.

**Table 1:** The energy densities of the ordinary St–PBS hybrid, St–PBS micelle I, St–PBS micelle II and St–PBS micelle III, calculated from the CV curves obtained from a two-electrode configuration (symmetrical cell), and compared with the three electrode measurements

	Scan rate (mV s^−1^)	200	100	50	20	10	5
Three electrode configuration	St–PBS (W h kg^−1^)	10	19	29	45	57	73
	St–PBS micelle I (W h kg^−1^)	12	21	35	47	61	79
	St–PBS micelle II (W h kg^−1^)	18	31	60	95	118	143
	St–PBS micelle III (W h kg^−1^)	11	21	32	46	59	77
Two electrode configuration (symmetrical cell)	St–PBS (W h kg^−1^)	21	31	35	37	42	45
	St–PBS micelle I (W h kg^−1^)	24	34	39	44	45	50
	St–PBS micelle II (W h kg^−1^)	25	40	44	52	63	67
	St–PBS micelle III (W h kg^−1^)	24	34	38	42	45	48

#### 3.4.2. Electrochemical measurements on starch–PBS sample using a two electrode configuration

**Fig. 6:**
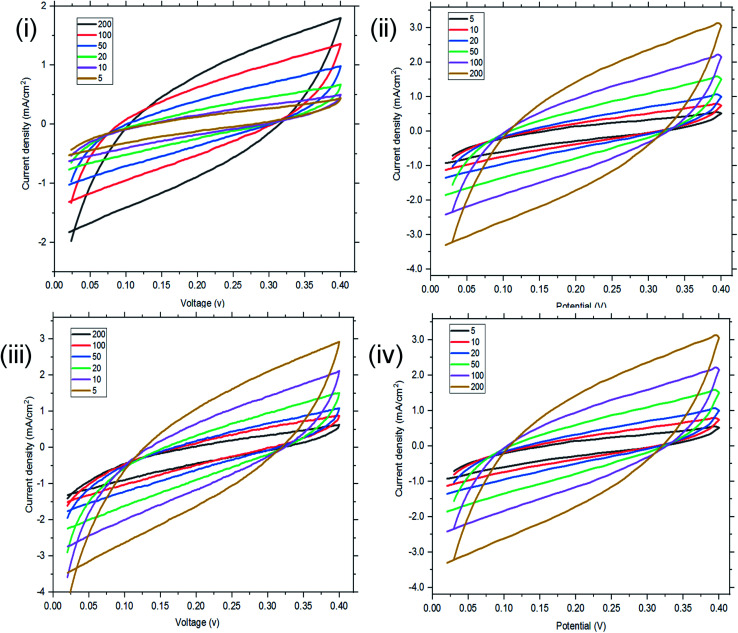
Two-electrode configuration (I–IV): CV of ordinary St–PBS hybrid, St–PBS micelle I, St–PBS micelle II and St–PBS micelle III.

The materials were then tested using a two-electrode cell configuration. For the two electrode configuration, a symmetrical cell supercapacitor was fabricated using the St–PBS micelles I, II and III with a glass fibre separator and the same electrolyte as was used with the three electrode configuration. The CV's, EIS, and GCD were then measured and *C*_sp_ and retention capacity were determined. The CV's were recorded from 0.0 to 0.4 V using 1 M Na_2_SO_4_ electrolyte, at scan rates of 5, 10, 20, 50, 100 and 200 mV s^−1^, and are presented in Fig. 4. The St–PBS micelle I had a *C*_sp_ of 101, 126, 144, 152, 170, and 182 F g^−1^. At the same scan rates, St–PBS micelle II showed *C*_sp_ of 106, 140, 159, 181, 185, 203 F g^−1^, and St–PBS micelle III showed *C*_sp_ of 103, 137, 155, 172, 183, 194 F g^−1^. These specific capacitances are lower than the ones reported for three-electrode configuration experiment (see Table S1† for comparison), but the quasi-rectangular shapes were retained, and confirms the supercapacitive properties of the micelles under the two-electrode configuration. The CV curves (Fig. 6) illustrate that the micelles store charges *via* a mechanism that is free from redox disturbances, oxygen reduction and electrolyte decomposition.

**Fig. 7:**
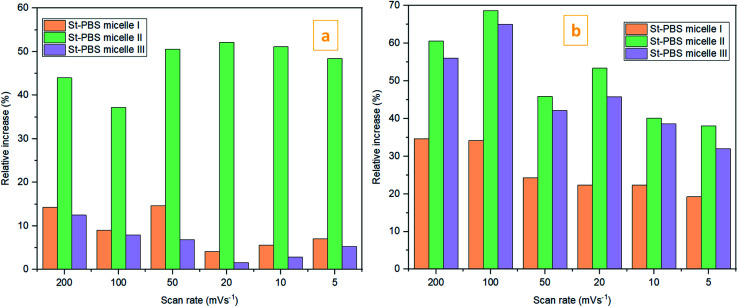
The relative increase in *C*_sp_
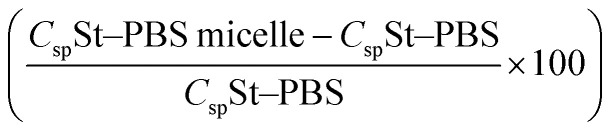
 for the St–PBS micelle samples when tested in a two-electrode configuration (A), *versus* the three-electrode symmetrical cell (B).

In the two-electrode configuration, the St–PBS micelle III had higher *C*_sp_ than St–PBS micelle I and St–PBS micelle II had the highest *C*_sp_ among the three samples (Table S1†). The relative increase, as a percentage, in *C*_sp_ of the micelle samples *versus* the ordinary St–PBS sample are presented in Fig. 7. St–PBS micelle II had the best performance in both systems of two and three electrode configuration, and this can be attributed to the morphology of the sample (see Fig. 1).

The relatively open porous structure seems to favour ingress of the electrolyte, over a possibly larger surface area, and allow for improved capacitive behaviour (kinetics and storage of charge) with the St–PBS micelle II sample.

**Fig. 8:**
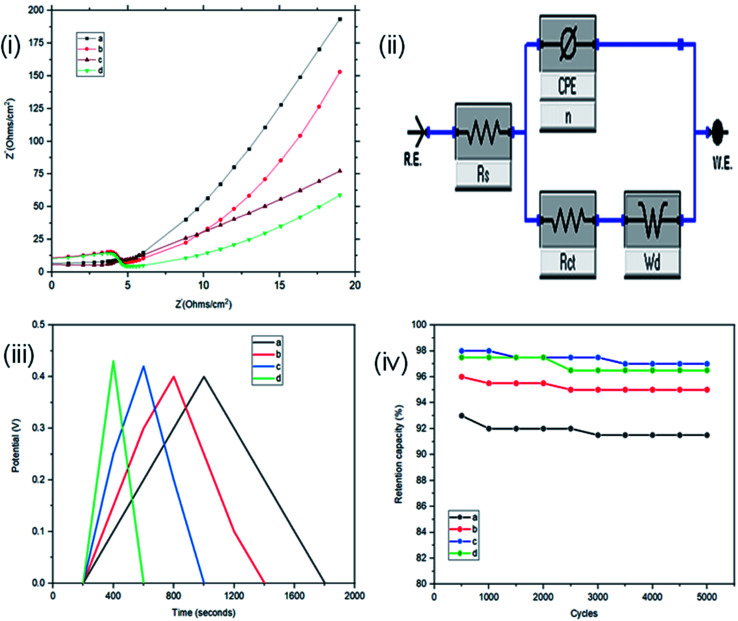
Two-electrode configuration: (i) EIS of ordinary St–PBS hybrid, St–PBS micelle I, St–PBS micelle II and St–PBS micelle III (a–d). (ii) Constant phase element (CPE); equivalent circuit model of the EIS (iii) GCD of ordinary St–PBS hybrid, St–PBS micelle I, St–PBS micelle II and St–PBS micelle III (a–d). (iv) Retention capacity of ordinary St–PBS hybrid, St–PBS micelle I, St–PBS micelle II and St–PBS micelle III (a–d).

The interface behaviour of the two electrode configuration for the supercapacitor materials were studied using EIS with a frequency range of 0.1 to 100 kHz, at an amplitude of 10 mV. From Fig. 6(i), the arc in the high frequency range indicates the charge transfer resistance and double layer capacitance that develops between the contact interface of the St–PBS micelles and the glassy fibre separator. The ‘*n*’ values of 0.690, 0.74 and 0.70 were obtained; while an equivalent series resistance (ESR) of 1.97, 2.92, and 2.90 ohms were obtained for St–PBS micelles I, II and III respectively. *R*_ct_ of 19.32, 18.17 and 19.08 ohms (see Fig. 6(ii) for model used) were obtained for the three respective starch based symmetrical supercapacitors. The resistances increased in the two-electrode configuration, the ‘*n*’ value increased and the supercapacitive parameters, which include specific capacitance, energy density and power density, decreased, when compared to the three-electrode configuration. GCD curves (Fig. 6(iii)) in the two-electrode configuration showed symmetrical and triangular shapes, indicating a good supercapacitive behaviour. The period of charge–discharge, cycles were 1040, 720 and 645 seconds for the St–PBS micelles I, II, III supercapacitor system. The three respective supercapacitors showed retention capacities (Fig. 8(iv)) of 97.0, 96.5 and 95.5%. Before micellization, the supercapacitor system of the ordinary St–PBS showed a retention capacity of 91.5%.

**Fig. 9:**
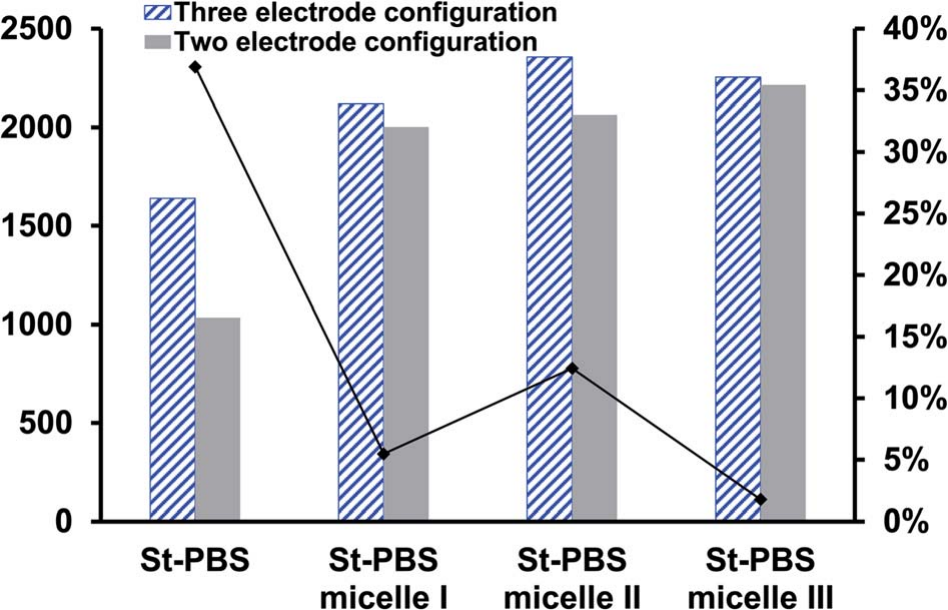
The power densities of the ordinary St–PBS hybrid, St–PBS micelle I, St–PBS micelle II and St–PBS micelle III, calculated from the data obtained with a three- and two-electrode configuration (symmetrical cell), and a comparison of the percentage decrease between corresponding samples in a three- and two-electrode configuration.

When compared with past works, the energy and power densities obtained in this work are very promising. This is because of effective diffusion and flow of radical cations within the micelles. The highest energy densities were obtained at the lowest scan rate of 5 mV s^−1^ used. After fabricating the micelles into symmetrical supercapacitors, St–PBS micelle I had an energy density of 50 W h kg^−1^, St–PBS micelle II showed energy density of 67 W h kg^−1^, and St–PBS micelle III had the lowest energy density of 48 W h kg^−1^ (Table 1). The three reverse micelle supercapacitor system, St–PBS micelle I, II and III had power densities of 2002, 2063, and 2214 W kg^−1^. The change in power densities when testing the materials in a three electrode configuration *versus* a two electrode configuration are summarised in Fig. 9.

Before micellization, power densities drops by 37%, while the decrease observed with St–PBS micelle I, II, and III were 5, 12 and 2% respectively. This further highlights another advantage of the micellization process; specifically, retention of the improved capacitive performance when switching between configurations. The starch micelle based supercapacitor system relies on the fast cation diffusion of the PBS backbone. The PBS forms the core of the reverse micelles and help to prevent ionic leakages, impedance surge, unstable kinetics and electrochemical irreversibility, which are the common shortcomings of biopolymers, towards energy storage applications.

### 3.5. Incorporation of magnetite

Magnetite nanoparticles have interesting physical–chemical properties that can be exploited to enhance the energy densities of the St–PBS micelles. They have multiple redox states, high thermal conductivity, and shows sharp conductance transitions.^63^ The FTIR of the starch based reverse micelles after magnetite incorporation are presented in Fig. S1.† The double peaks at 548 and 440 cm^−1^ indicate the successful incorporation of the magnetite nanoparticles. Fe–O–H peak was seen at 1433 cm^−1^, indicating that the magnetite nanoparticles are stabilized by the starch micelles.^64^ The C–H peaks of the St–PBS micelle III with magnetite (St–PBS-III–Fe_3_O_4_) were more intense, than the C–H peaks found in St–PBS micelle I with magnetite (St–PBS-I–Fe_3_O_4_) and the St–PBS micelle II with magnetite (St–PBS-II–Fe_3_O_4_). Carbonyl peak at 1635 cm^−1^ confirms the presence of starch biopolymers with reduced end groups, and C–O–C bond of the starch was observed at 1055 cm^−1^.^65^ The XRD patterns (Fig. S1(ii)†) also confirmed the presence of Fe_3_O_4_ nanoparticles.^66^ The peak at 2*θ* of 37° indicates the (311) crystal plane of magnetite nanoparticles, while the diffraction peaks at 2*θ* = 19.1° and 24.3° indicates the crystalline structure of starch biopolymers. The peak at 42.5° shows the (400) crystal planes of gamma magnetite nanoparticles, which are obtained at low temperature conditions.

The SEM images of the three magnetite incorporated reverse micelles showed similar morphologies (Fig. S2†). The St–PBS–Fe_3_O_4_ micelles appeared as agglomerated particles, with irregular spherical morphology. The morphology observed was similar to what has been reported in the literature.^67–69^

**Fig. 10:**
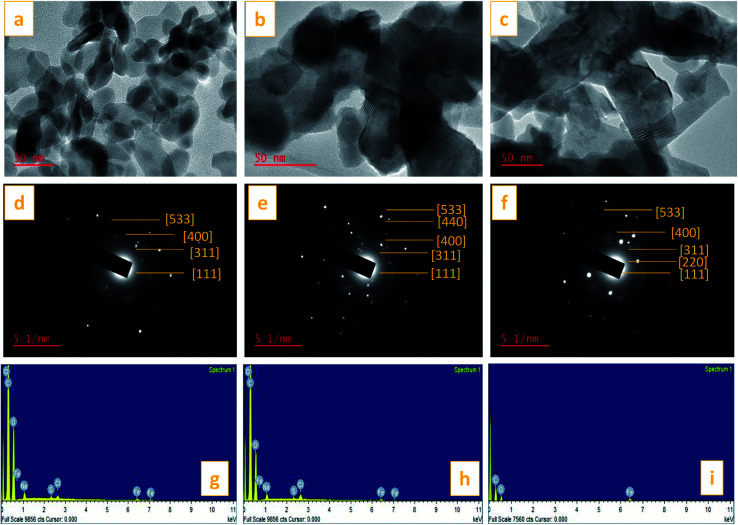
Images (a)–(c) are TEM micrographs of St–PBS-I–Fe_3_O_4_, St–PBS-II–Fe_3_O_4_ and St–PBS-III–Fe_3_O_4_. Images (d)–(f) are SAED patterns of St–PBS–Fe_3_O_4_-I, St–PBS–Fe_3_O_4_-II and St–PBS–Fe_3_O_4_-III. Images (g)–(i) EDX of St–PBS–Fe_3_O_4_-I, St–PBS–Fe_3_O_4_-II and St–PBS–Fe_3_O_4_-III.

TEM images of the St–PBS micelles with the magnetite incorporated are presented in Fig. 10. The magnetite formed spherical nanoparticles which coated the starch reverse micelles in St–PBS-I–Fe_3_O_4_, while, the magnetite nanoparticles appeared as dispersions in the St–PBS-II–Fe_3_O_4_ and St–PBS-III–Fe_3_O_4_ samples. Selected Area Electron Diffraction (SAED) showed crystalline ring patterns, which were indexed with respect to inter-planar spacing of incorporated magnetite nanoparticles. Besides the (311) and (400) crystal phases, which were also observed with the XRD analysis, the SAED pattern for the St–PBS-I–Fe_3_O_4_ had rings indexed at (533), and (111), the St–PBS-II–Fe_3_O_4_ had an additional ring indexed to (220), and the St–PBS-III–Fe_3_O_4_ showed a ring indexed to (440) crystalline phase for magnetite. Similar results have been reported in the literature for magnetite starch composites.^70^ The Energy Dispersive X-ray (EDX) results confirmed that the three magnetite incorporated reverse micelles contained C, O and Fe with other minor peaks like Na, Cl and S peaks, from *p*-toluene sulphonic acid activation treatments.

### 3.6. Electrochemical measurements on starch–PBS reverse micelles samples incorporated with magnetite

**Fig. 11:**
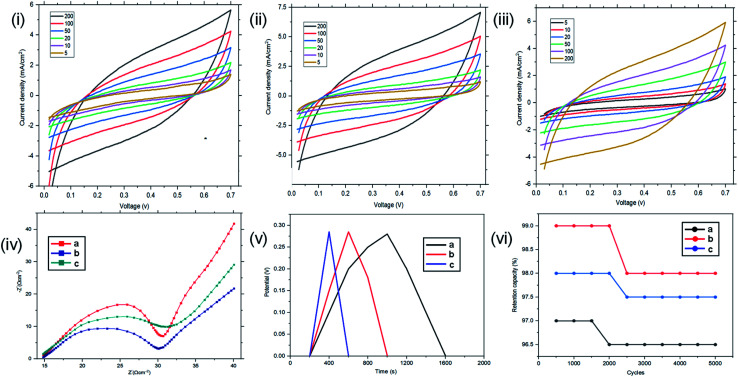
Results using a three-electrode configuration for the CV of St–PBS–Fe_3_O_4_-I (i), St–PBS–Fe_3_O_4_-II (ii) and St–PBS–Fe_3_O_4_-III (iii). The EIS (iv) of St–PBS–Fe_3_O_4_-I, St–PBS–Fe_3_O_4_-II and St–PBS–Fe_3_O_4_-III (curves a–c respectively). The GCD curves (v) of the St–PBS–Fe_3_O_4_-I, St–PBS–Fe_3_O_4_-II and St–PBS–Fe_3_O_4_-III (curves a–c respectively). The retention capacity (vi) of the St–PBS–Fe_3_O_4_-I, St–PBS–Fe_3_O_4_-II and St–PBS–Fe_3_O_4_-III samples (curves a–c respectively).

The samples with magnetite nanoparticles incorporated into the reverse micelles were then investigated using CV, EIS and GCD using both two and three electrode configurations. CV results using the three electrode configurations are presented in Fig. 11. In the three-electrode configuration, at scan rates of 200, 100, 50, 20, 10, and 5 mV s^−1^, St–PBS-I–Fe_3_O_4_ had *C*_sp_ of 86, 173, 288, 435, 527, and 608 F g^−1^, while St–PBS-II–Fe_3_O_4_ had *C*_sp_ of 108, 172, 309, 442, 532, and 631 F g^−1^ respectively. The third magnetite incorporated micelle, St–PBS-III–Fe_3_O_4_ had *C*_sp_ of 79, 154, 278, 393, 484, and 589 F g^−1^. Similar trends seen with the St–PBS micelle samples before magnetite incorporation were observed with samples with magnetite. At a scan rate of 5 mV s^−1^, the highest *C*_sp_ values were observed, and the relative increases were (at 5 mV s^−1^) 87.7, 8.05, and 85.2% for the St–PBS micelle I, II, and III samples before and after magnetite incorporation. The increase in *C*_sp_ values could be attributed to some changes in morphology, as noted with SEM images, and an increase in charge storage sites due to the interfaces between the magnetite nanoparticles and the St–PBS micelle hybrids.

The increase in *C*_sp_ observed with our work is greater than some values reported in the literature. For example, in the work reported by Rhadakrishan, where polypyrrole–magnetite and polyaniline–magnetite were designed, the largest *C*_sp_ reported was 210 F g^−1^.^71^ Wang *et al.*, obtained a *C*_sp_ of 220 F g^−1^ after he incorporated magnetite nanoparticles in graphene, Oh *et al.*, achieved an increase in *C*_sp_ from 99.4 to 202 F g^−1^ after magnetite incorporation.^31,72^

As shown in Table 2, at scan rate of 5 mV s^−1^, the three magnetite incorporated St–PBS reverse micelles had energy densities of 148, 154 and 146 W h kg^−1^. The aim of magnetite incorporation is to improve the energy density of the starch based micelles. The relative increase in energy densities of the reverse micelles after magnetite nanoparticle incorporation was 87.3, 7.7, and 89.6% for the St–PBS micelle I, II, and III respectively. Energy density shows how much energy a supercapacitor electrode can store, and it provides an indication on where the supercapacitor can be applied.

**Table 2:** The energy densities for the magnetite incorporated St–PBS micelle I, St–PBS micelle II and St–PBS micelle III, calculated from the CV curves obtained from a two-electrode configuration (symmetrical cell), and compared with the three electrode measurements

	Scan rate (mV s^−1^)	200	100	50	20	10	5
Three electrode configuration	St–PBS-I–Fe_3_O_4_ (W h kg^−1^)	21	42	70	106	129	148
	St–PBS-II–Fe_3_O_4_ (W h kg^−1^)	26	42	75	108	130	154
	St–PBS-III–Fe_3_O_4_ (W h kg^−1^)	20	36	67	102	119	146
Two electrode configuration (symmetrical cell)	St–PBS-I–Fe_3_O_4_ (W h kg^−1^)	26	40	45	53	64	66
	St–PBS-II–Fe_3_O_4_ (W h kg^−1^)	30	41	53	64	72	79
	St–PBS-III–Fe_3_O_4_ (W h kg^−1^)	27	39	49	59	67	68

Due to the high energy densities of these magnetite incorporated starch micelles, some potential areas of application include electro-rheological fluids, and in LED power sources. Finally, the St–PBS-I–Fe_3_O_4_ had a power density of 2118 W kg^−1^, the St–PBS-II–Fe_3_O_4_ had a power density of 4371 W kg^−1^, and the St–PBS-III–Fe_3_O_4_ had a power density of 2356 W kg^−1^. Sevilla *et al.*, reported a *C*_sp_ of 200 F g^−1^ after activation of biomass with sodium sulphate; Sudhakar and Kumar,^73^ obtained a *C*_sp_ of 115 F g^−1^ for starch doped with poly(styrene sulfonic acid), while Han *et al.*,^74^ reported a volumetric capacitance of 584 F cm^−3^ for starch activated with poly(4-styrene sulfonate). In our method, we employed *para* toluene sulfonic acid and propylene carbonate in the presence of chloride ions to activate our starch based micelles. Our work achieved specific capacitance and energy density values, which are greater than the obtainable values in current literatures, for starch based supercapacitors.

**Fig. 12:**
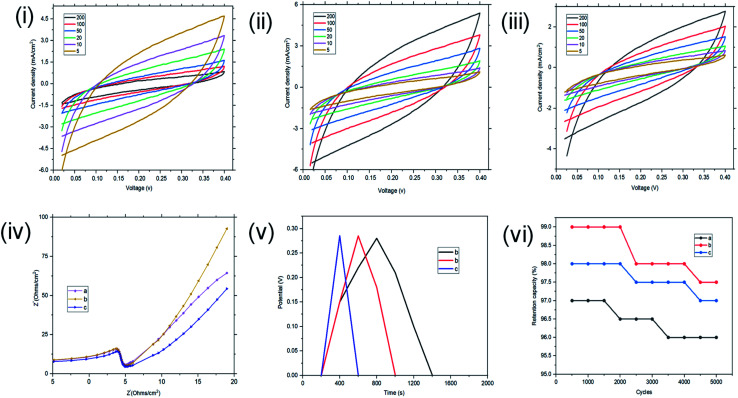
Using a two-electrode configuration, the CV data in images I–III are St–PBS–Fe_3_O_4_-I, St–PBS–Fe_3_O_4_-II and St–PBS–Fe_3_O_4_-III respectively. The image in panel IV is the EIS data of St–PBS–Fe_3_O_4_-I, St–PBS–Fe_3_O_4_-II and St–PBS–Fe_3_O_4_-III (labelled as a–c respectively). The image labelled (V) is the GCD data of St–PBS–Fe_3_O_4_-I, St–PBS–Fe_3_O_4_-II and St–PBS–Fe_3_O_4_-III (samples labelled a–c respectively). Finally, (VI) is the retention capacity of St–PBS–Fe_3_O_4_-I, St–PBS–Fe_3_O_4_-II and St–PBS–Fe_3_O_4_-III (labelled a-c respectively).

In the two-electrode configuration, the key features in terms of shapes or symmetry of the curves during CV and GCD experiments did not change significantly, indicating the ideal capacitive behaviour of the materials before (see Fig. 6) and after magnetite incorporation (Fig. 12). At scan rates of 200, 100, 50, 20, 10, 5 mV s^−1^, the St–PBS-I–Fe_3_O_4_ had *C*_sp_ of 107, 164, 183, 237, 277, and 273 F g^−1^; similarly, St–PBS-II–Fe_3_O_4_ showed *C*_sp_ values of 124, 168, 218, 263, 295, and 308 F g^−1^ and the St–PBS-III–Fe_3_O_4_ had *C*_sp_ of 109, 160, 202, 241, 284, and 291 F g^−1^. The energy density of the three respective micelles at 5 mV s^−1^ were 66, 79, 68 W h kg^−1^; and the power densities were 3118, 3291, 3157 W kg^−1^. The values for *C*_sp_ and *E*_d_ for the two- and three-electrode configuration are summarised and compared in Tables S2† and 2. The high energy and power density achieved is due to effective diffusion of polarons in the conductive starch reverse micelles, incorporated with magnetite nanoparticles. The period of charge and discharge shown on the GCD curves of the magnetite incorporated micelles were 460, 435 and 320 seconds respectively. The high energy density achieved is an indication that magnetite nanoparticles aid the flow of charges within the reverse micelles. The St–PBS-I–Fe_3_O_4_ had a retention capacity of 96.0%, the St–PBS-II–Fe_3_O_4_ had 97.5% and St–PBS-III–Fe_3_O_4_ had 97.0%, after 5000 cycles (Fig. 12(vi)).

## 4. Conclusions

A new route, which is cost effective and environmentally friendly, was employed to design conductive un-carbonized starch for energy storage. Three different methods were employed to make three reverse micelles of St–PBS hybrids, the PBS providing strength and higher operational temperature window for the hybrid reverse micelles. The reverse micellar starch hybrid prepared using a single emulsion technique, St–PBS micelle II, produced agglomerated structures with some porosity that could account for the excellent capacitive properties observed. The St–PBS micelle II had the highest *C*_sp_ of 584 F g^−1^ and 203 F g^−1^, and energy densities of 143 and 67 W h kg^−1^ in a three and two electrode configuration. The 2–12% drop in power densities for the reverse micelle samples when changing from a three to a two-electrode set-up (symmetrical super capacitor) shows the materials have excellent potential for scale-up and device assembly.

Magnetite nanoparticles were incorporated into the micelles to achieve a cycling stability of 98% after 5000 cycles, a maximum *C*_sp_ of 631 (three-electrode test) F g^−1^ and 308 F g^−1^ (two electrode testing), and *E*_d_ of 154 (three electrode test) and 79 (two electrode test) W h kg^−1^. The significance with the values reported in this work is that all the samples were in un-carbonized natural polymers hybrids. Furthermore, this research showed that different micellar designs exhibit different ionic and electronic path properties leading to variations in conductivity and capacitance. Thus offering new routes in the design and implementation of natural un-carbonised polymers for energy storage. The reverse micelle II, which was designed using a single emulsion technique, showed the best supercapacitive properties in both two and three cells configurations. This is attributed to the unique stable architecture, and enhanced surface and charge properties. The constant phase element (CPE) which was used to model the surface kinetics of the St–PBS based micelles showed that they are good platforms for supercapacitive energy storage. Non-toxic, chemically stable, affordable and available materials were used throughout this work. The findings will provide alternatives in the science and engineering of cleaner and sustainable energy storage systems. Thus, with our approach, un-carbonized biopolymers can contribute to meeting the United Nations 7th sustainable development goal of ‘Affordable and Clean Energy’.

## Conflicts of interest

There are no conflicts to declare.

## Supplementary Material

RA-011-D1RA00635E-s001
