# Supplementary material for: Micellization of a starch–poly(1,4-butylene succinate) nano-hybrid for enhanced energy storage
Source: RSC Adv. 2021 Mar 22;11(19):11745–59. doi: 10.1039/d1ra00635e (PMC8695992; doi:10.1039/d1ra00635e)
Supplement: RA-011-D1RA00635E-s001 [file RA-011-D1RA00635E-s001.pdf]

Electronic Supplementary Material (ESI) for RSC Advances.
This journal is © The Royal Society of Chemistry 2021

Micellization of starch- poly (1,4-butylene succinate) nano-hybrid for enhanced energy storage.

O.D. Saliu, M. A Mamo*, P. G. Ndungu*, and J. Ramontja*

Energy, Sensors and Multifunctional Nanomaterials Research Group, Department of Chemical Sciences, University of Johannesburg, P.O. Box 17011, Doornfontein 2028 Johannesburg, South Africa

*Corresponding Authors: messaim@uj.ac.za, jamesr@uj.ac.za; pndungu@uj.ac.za

Supplementary Information

The specific capacitance, C_{sp}, was calculated using the following equation[1,2]:

$$C_{sp} = \frac{I\Delta V}{2mv\Delta V}$$

Where $I\Delta V$ is obtained as the area of the CV curve, m is the mass of active material loaded, v is the scan rate in mVs^{-1}, ΔV is the potential window.

The energy density (E_d) was calculated using the following equation[1,2]:

$$E_d = \frac{1}{2} C_{sp} V^2$$

Where C_{sp} is the specific capacitance and V is the nominal voltage.

Finally, the power density, P_d, was calculated using the following equation[1,2]:

$$P = \frac{V^2}{4ESR * m}$$

Where V is the nominal voltage, ESR is the equivalent series resistance, and m is the mass of active material loaded.

Table S1: The specific capacitances of the ordinary St-PBS hybrid, St-PBS micelle I, St-PBS micelle II and St-PBS micelle III, calculated from the CV curves obtained from a two-electrode configuration (symmetrical cell), and compared with the three electrode measurements.

	**Scan rate (mV/s)**	**200**	**100**	**50**	**20**	**10**	**5**
**Three electrode configuration**	**St-PBS (C_{sp}/ Fg^{-1})**	42	81	122	187	236	301
	**St-PBS Mic I (C_{sp}/ Fg^{-1})**	49	89	143	195	250	324
	**St-PBS Mic II (C_{sp}/ Fg^{-1})**	75	129	247	391	483	584
	**St-PBS Mic III (C_{sp}/ Fg^{-1})**	48	88	131	190	243	318
**Two electrode configuration (Symmetrical Cell)**	**St-PBS (C_{sp}/ Fg^{-1})**	66	83	109	118	132	147
	**St-PBS Mic I (C_{sp}/ Fg^{-1})**	101	126	144	152	170	182
	**St-PBS Mic II (C_{sp}/ Fg^{-1})**	106	140	159	181	185	203

	**St-PBS Mic III (C_{sp}/ Fg^{-1})**	103	137	155	172	183	194

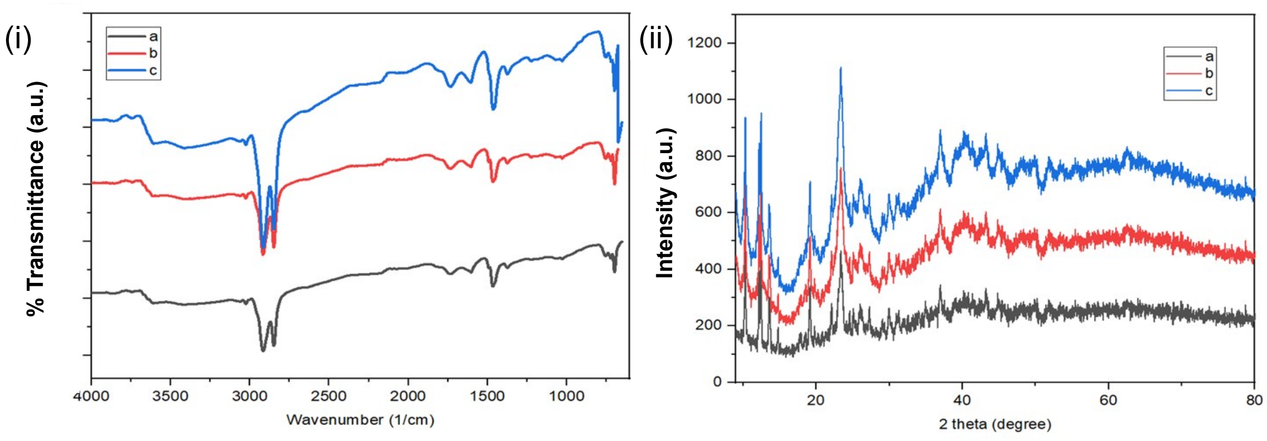

Figure S1: Image (i) presents the FTIR data and image (ii) are the XRD patterns for the St-PBS-I-Fe_3O_4, St-PBS-II-Fe_3O_4, and St-PBS-III-Fe_3O_4.

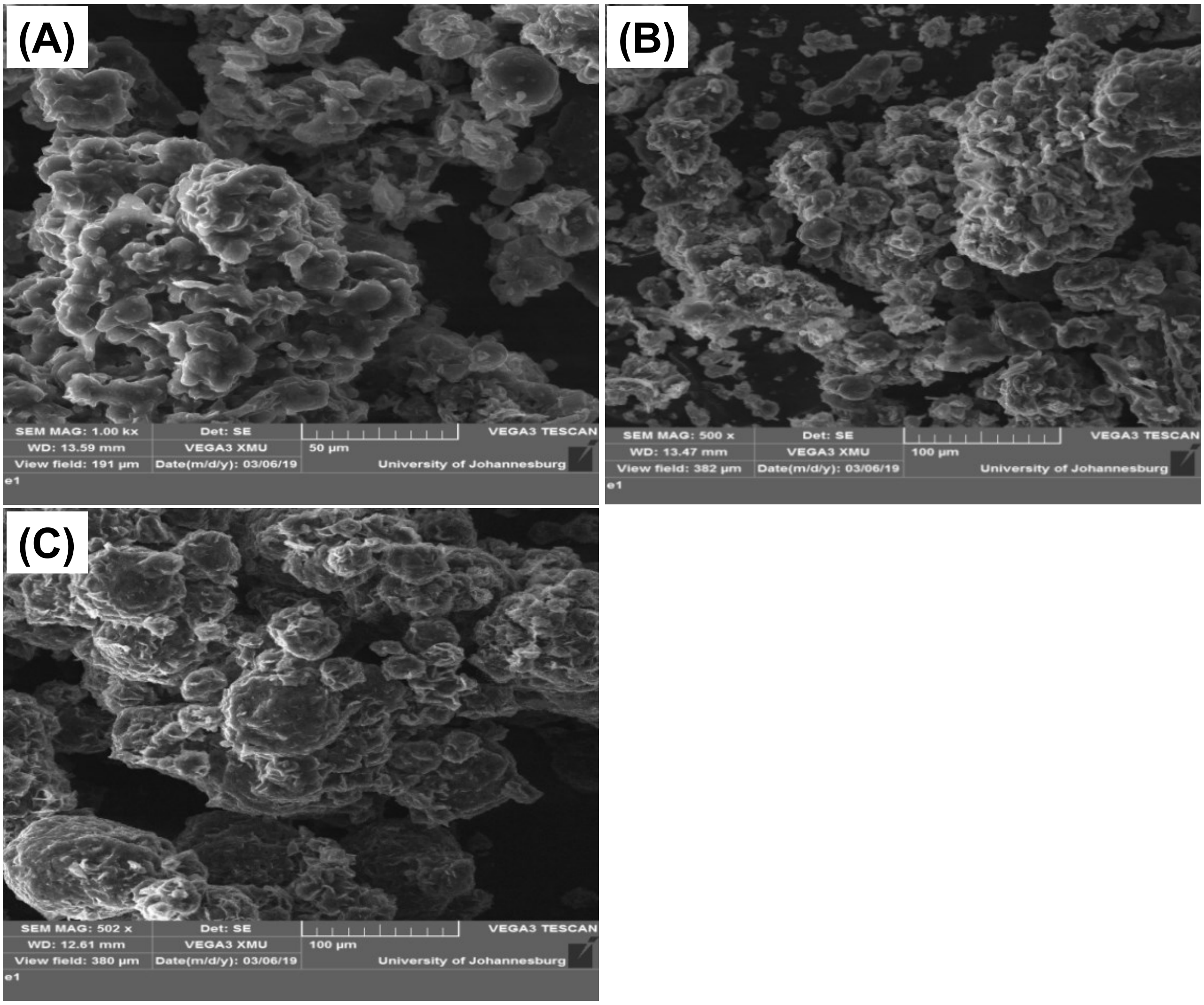

Figure S2: SEM images of the (a) St-PBS-I-Fe_3O_4, (b) St-PBS-II-Fe_3O_4, and (c) St-PBS-III-Fe_3O_4 samples.

Table S2: The specific capacitances of the magnetite incorporated St-PBS micelle I, St-PBS micelle II and St-PBS micelle III, calculated from the CV curves obtained from a two-electrode configuration (symmetrical cell), and compared with the three electrode measurements.

	**Scan rate (mV/s)**	**200**	**100**	**50**	**20**	**10**	**5**
**Two electrode configuration (Symmetrical Cell)**	**St-PBS-I-Fe_3O_4 (C_{sp}/ Fg^{-1})**	107	164	183	237	277	273
	**St-PBS-II-Fe_3O_4 (C_{sp}/ Fg^{-1})**	124	168	218	263	295	308
	**St-PBS-III-Fe_3O_4 (C_{sp}/ Fg^{-1})**	109	160	202	241	284	291
**Three electrode configuration**	**St-PBS-I-Fe_3O_4 (C_{sp}/ Fg^{-1})**	86	173	288	435	527	608
	**St-PBS-II-Fe_3O_4 (C_{sp}/ Fg^{-1})**	108	172	309	442	532	631
	**St-PBS-III-Fe_3O_4 (C_{sp}/ Fg^{-1})**	79	154	278	393	484	589

References

1 Y. Ko, M. Kwon, W. K. Bae, B. Lee, S. W. Lee and J. Cho, *Nat. Commun.*, 2017, **8**, 536.

2 D. Ghosh, S. Giri, M. Moniruzzaman, T. Basu, M. Mandal and C. K. Das, *Dalt. Trans.*, 2014, **43**, 11067–11076.
